# Biomechanical phenotyping pipeline for stalk lodging resistance in maize

**DOI:** 10.1016/j.mex.2024.102562

**Published:** 2024-01-09

**Authors:** Kaitlin Tabaracci, Norbert T. Bokros, Yusuf Oduntan, Bharath Kunduru, Joseph DeKold, Endalkachew Mengistie, Armando McDonald, Christopher J. Stubbs, Rajandeep S. Sekhon, Seth DeBolt, Daniel J. Robertson

**Affiliations:** a Department of Mechanical Engineering, University of Idaho, Moscow, ID, USA; b Department of Horticulture, University of Kentucky, Lexington, KY, USA; c Department of Genetics and Biochemistry, Clemson University, Clemson, SC, USA; d Department of Forest, Rangeland and Fire Sciences, University of Idaho, Moscow, ID, USA; e School of Computer Sciences and Engineering, Fairleigh Dickinson University, Teaneck, NJ, USA

**Keywords:** *Biomechanical phenotyping pipeline for maize*, Maize, Stalk, Lodging, Phenotyping, Biomechanical, Bending strength, Bending stiffness

## Abstract

Stalk lodging (structural failure crops prior to harvest) significantly reduces annual yields of vital grain crops. The lack of standardized, high throughput phenotyping methods capable of quantifying biomechanical plant traits prevents comprehensive understanding of the genetic architecture of stalk lodging resistance. A phenotyping pipeline developed to enable higher throughput biomechanical measurements of plant traits related to stalk lodging is presented. The methods were developed using principles from the fields of engineering mechanics and metrology and they enable retention of plant-specific data instead of averaging data across plots as is typical in most phenotyping studies. This pipeline was specifically designed to be implemented in large experimental studies and has been used to phenotype over 40,000 maize stalks. The pipeline includes both lab- and field-based phenotyping methodologies and enables the collection of metadata. Best practices learned by implementing this pipeline over the past three years are presented. The specific instruments (including model numbers and manufacturers) that work well for these methods are presented, however comparable instruments may be used in conjunction with these methods as seen fit.

• Efficient methods to measure biomechanical traits and record metadata related to stalk lodging.
• Can be used in studies with large sample sizes (i.e., > 1,000).

## Graphical abstract

**Figure:**
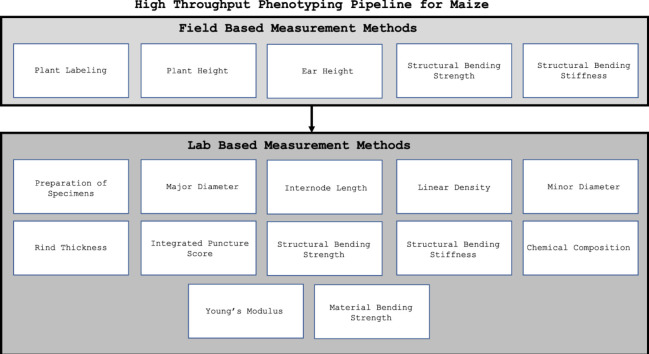

Specifications table

**Table:** 

Subject area:	Agricultural and Biological Sciences
More specific subject area:	Phenotyping Agri
Name of your method:	Biomechanical phenotyping pipeline for maize
Name and reference of original method:	Robertson, D.J., Cook, D.D., Meehan, K., Asatiani, L., 2020. The Effect of Probe Geometry on Rind Puncture Resistance Testing of Maize Stalks. https://doi.org/10.1186/s13007-020-00610-8 Robertson, D.J., Smith, S.L., Cook, D.D., 2015. On measuring the bending strength of septate grass stems. American Journal of Botany 102, 5–11. https://doi.org/10.3732/ajb.1400183 Robertson, D., Smith, S., Gardunia, B., Cook, D., 2014. An Improved Method for Accurate Phenotyping of Corn Stalk Strength. Crop Science 54, 2038. https://doi.org/10.2135/cropsci2013.11.0794 Seegmiller, W.H., Graves, J., Robertson, D.J., 2020. A novel rind puncture technique to measure rind thickness and diameter in plant stalks. Plant Methods 16, 44. https://doi.org/10.1186/s13007-020-00587-4 Stubbs, C.J., McMahan, C., Seegmiller W., Cook, D.D., Robertson, D.J., 2020. Integrated Puncture Score: force–displacement weighted rind penetration tests improve stalk lodging resistance estimations in maize. Plant Methods 16, 113. https://doi.org/10.1186/s13007-020-00654-w
Resource availability:	NA

## Method details

Phenotypes at numerous spatial scales contribute to stalk lodging. Methods for measuring phenotypes at the plant level (plant height, ear height, structural bending stiffness, and structural bending strength), organ level (major and minor diameter, rind thickness, internode length, linear density, rind penetration resistance and integrated puncture score) and tissue level (chemical composition, tissue bending strength and Young's modulus) are presented. The methodologies are presented below in the chronological order in which they are typically acquired.

## Plant labeling

The following best practices in experimental design were developed after growing and testing an association panel of maize for three years at two locations. To allow the collection of metadata and preservation of the identity of individual plant samples, all plants were individually labeled in the field with a unique identifier referred to as the StalkID. The StalkID included the location grown, the year of the study, the plot number, genotype information, and the stalk number. This information was printed in text on each plant label and embedded into a QR code that was placed on the label. QR codes were created using Bartender (Seagull Scientific, Bellevue, WA) at a 7% recovery level, in contrast to a higher default recovery level used by most QR code generating software packages. Using higher recovery levels frequently introduced barcode reading errors in one or more characters of the StalkID. This is especially problematic as incorrect barcode scans typically remain unnoticed and introduce large errors in data postprocessing. To enable automated checking for barcode reading errors, the QR code consisted of the StalkID repeated three times (i.e., “StalkID__StalkID__StalkID”). This redundancy enabled a simple algorithm to identify and correct any misread characters in the barcode. The StalkID's typically included the syntax “LocationYear_Plot-Stalk”. Since the StalkID is often used as a filename to store data on individual stalks, periods, slashes, asterisks, and other special characters should be avoided as these are prohibited filename characters. The labels which contained the QR codes were printed on waterproof and weather resistant synthetic polyester paper (SYNAPS XM 5 MIL sheets) (Nekoosa, Nekoosa, WI) and stapled to the plants at V10 plant growth stage. They were stapled using Ace Clippers (Model No. 702) (Ace, Oak Brook, IL). QR barcodes were easier to scan and less prone to barcode reading errors as compared to 1D barcodes.

## Plant height & ear height

For recording plant and ear height data, 20 feet long PVC pipes were converted into measuring sticks by marking length in inches on the pipes. The plant and ear height data were recorded on each plant one week after anthesis. To record the height data, the marked PVC pipes were aligned with the stalk of each plant that was phenotyped and the distance from the base of the stalk to the tip of the central spike of the tassel was recorded as plant height. Similarly, the distance from the base of the stalk to the primary-ear bearing node was recorded as the ear height.

## Field-based measurements of structural bending strength & structural bending stiffness

Structural bending strength is a key determinant of stalk lodging resistance [14]. A Device for Assessing Resistance to Lodging In Grains (DARLING) [1] was used to acquire structural bending strength and structural bending stiffness measurements on field-grown maize plants. The DARLING device consists of a force sensor attached to a vertical arm which is in turn connected to a hinged footplate. To use the device the operator places the device next to a stalk, aligns the stalk with the force sensor, steps on the footplate, and deflects the stalk by pushing on the device. Force and rotation are continuously recorded during the test and are used to calculate stalk flexural stiffness and stalk bending strength as described in [1]. Several experimental factors can significantly impact the accuracy of DARLING measurements (e.g., [2]). The best practices outlined below can be used to mitigate errors and ensure accurate and efficient data collection.

Since multiple DARLING devices are typically deployed for data collection for a single experiment, all devices were calibrated prior to testing to ensure repeatability and accuracy across devices. The load cell was calibrated by detaching it from the device and placing it on a flat surface so that weight could be applied in the direction of the reading. Five readings were taken by applying weights between 0 and 25 pounds. The IMU and rotary potentiometer sensors were calibrated using a digital angle and level gauge (TickTock Tools, Dallas,TX). Five readings were taken at angles ranging between 80 and 180°, which corresponds to the full range of motion of the device during data collection. These five readings for each sensor were then fitted to a nonlinear regression line using the devices onboard software which allows the calibration of each sensor. It is important to collect five points along the full range of the sensor so the regression can account for any non-linearities in the sensors.

Before testing, all ears and leaves were removed from the plant, and the stalk was cut in the middle of the internode immediately above the primary ear-bearing node. Removal of leaves and the top part of the stalk was done to prevent adjacent plants from interfering with the testing of the subject plant during deflection. Furthermore, the removal of the upper part of the stalk prevented the introduction of errors into the force measurements due to oscillation in the upper part of the stalk. This can be done because the overall height of the stalk does not directly affect the ultimate bending strength of the stalk or of the root-soil complex and the effect of the self-supporting interacting leaves is negligible as the strength of the stalk is much higher than that of the interacting leaves [16]. Additionally, the plant weight has a negligible effect on stiff and strong stems like maize and bamboo [19]. This practice significantly reduced errors and provided clean and reproducible data.

Prior to commencing each test, the height of the load cell on the DARLING was adjusted so that it contacted the stalk just below the ear-bearing node. However, since most stalks within a plot are of similar height, the user adjusted the height of the device to the shortest plant of a plot and maintained this height for testing all the plants in the plot. Avoiding adjusting the height for each individual plant can save a tremendous amount of time when testing thousands of stalks. However, there is a slight tradeoff in accuracy for speed if the load cell height is set once for the whole plot versus adjusting per plant to account for ear height. In other words for some of the plants in the plot the load cell contacted the stalk at the second internode below the primary ear bearing node.

Prior to starting each test, care was taken to ensure the pivot point on the base of the DARLING (i.e., the hinge of the footplate) was as close as possible to the base of the stalk being tested, and that the vertical arm and load cell of DARLING was properly aligned with the stalk. Placement of the DARLING either in front of or behind the stalk introduces experimental error in force and angular deflection measurements [2].

To initiate a test the user utilizes the graphical user interface of the DARLING to select “START TEST”. At this point, the user is prompted to scan the QR code on the label of the plant being tested. The user would then use a small 2D wireless barcode scanner with Bluetooth connectivity (e.g., model EY-016–5pcs, Eyoyo, Guangdong, China) worn like a ring on the user's finger to scan the QR code on the plant label. The barcode scanner ensures quick and accurate entry of the StalkID and prevents human error caused by manual data input with a keyboard. After scanning the QR code, the StalkID was displayed on the DARLING screen, and the user had the option to accept or reject the scanned StalkID. Manual entry was only used if the barcode was unreadable due to damage caused by weather elements. Plants were labeled and tested sequentially and chronologically within each plot to provide an extra layer of protection against incorrect barcode scans or errors in data entry. Because the DARLING is equipped with a real-time clock the timestamps and testing order allowed for automated checking of StalkID and assisted in data correction or recovery during post-processing if necessary.

The test commenced in the following fashion. First, the DARLING was pushed slowly at a constant rate, to deflect the plant by 5–10° after which the DARLING and stalk were returned to their original upright position. This process was repeated three times. On the fourth push, the stalk was deflected until structural failure occurred resulting in either breakage (snap) or buckling of the stalk, indicating stalk lodging, or dislodging of the plant at the soil surface, signifying root lodging [9]. The user could then append a note to each tested stalk using the graphical user interface on the DARLING device specifying if the plant root lodged, stalk lodged, or simply bent over but did not break. The user ensured the DARLING and the stalk being tested did not come into contact with neighboring plants or with the ground as contact with external objects introduces significant errors in force measurements. The time of day at which DARLING tests were performed affected the measurements. In particular, the presence of dew during the early morning resulted in high soil moisture, decreased the strength of the root-soil complex, and caused a higher percentage of plants to root lodge. Thus, testing should begin after the soil has sufficiently dried. In addition, when testing in early morning if the plant did stalk lodge, then the failure type was often different than those observed later in the day. In the morning turgor pressure is high and tends to result in the stalks snaping in two rather than exhibiting the typical failure pattern for the stalk to buckle by forming a crease just above a node [9].

Data from the first three pushes in which the plant was deflected by 5 to 10° were used to determine the structural bending stiffness of the plant. Data from the fourth push in which the plant was deflected until failure was used to determine the structural bending strength of the plant. Recorded data includes angle of rotation, force, elapsed time, and GPS coordinates. DARLING devices can also be outfitted with humidity and temperature sensors in which case these data are also recorded for each test. Each test is stored in a structured CSV file. The header of the CSV file contains metadata, sensor calibration values, test attributes and optional data such as field notes. The body of the CSV file contains time, force, and displacement data. Post processing of these data files enables calculation of bending strength and flexural stiffness. Bending strength (maximum applied bending moment) is calculated by finding the maximum bending moment, *M* at failure:

(1) M=Fh

where *F* is the applied force and *h* is the height of the load cell (height of applied force). Flexural stiffness, *EI*, is calculated using the deflection equation of a cantilever beam:

(2) $$EI=\frac{\emptyset h^{3}}{3}$$

where ∅ is the slope of the linear portion of the force deflection curve and *h* is the height of the load cell (height of applied force). Deflection is calculated as:

(3) Deflection=hsin(θ)

where θ is the rotational displacement measured by the DARLING [1] and *h* is the height of the load cell (height of applied force).

These equations take into consideration several engineering assumptions which are explained in detail in [1]. It is assumed that the load cell remains perpendicular to the stalk throughout the duration of the test. The equations also assume small angle deflections & small strains.

## Laboratory‐based testing for various intermediate traits

### Preparation of specimens

After testing with the DARLING, the stalks were removed from the field for further testing in the lab. The stalks were cut at ground level using a pair of garden shears or loppers and any remaining leaves were removed. Care was taken to ensure the barcode tag was still firmly attached to the stalk. If the stalk broke into two pieces, the halves were adjoined together with Velcro. Stalks from a single plot were placed in a burlap bag, and the bag was labeled with the row tag carrying the plot number in numerical and barcode format. All stalks were dried in the greenhouse with a temperature maintained at 100°F and the fans running continuously to remove moisture from the air for six weeks. The burlap bags were spread out evenly throughout the greenhouse. Care was taken to avoid stacking bags on top of one another, and the bags were rotated twice a week to ensure even drying of all stalks in a bag. Depending on location, relative humidity, and the amount of moisture in the stalk at harvest, the temperature and drying time may need to be altered. It is very important that the drying is done gradually at low temperature coupled with proper air circulation as quick drying at high heat leads to geometric deformation of the stalks and confounds the lab analyses. Conversely, if the drying process takes too long or if there is not enough airflow, the stalks begin to get infected with mold and can start rotting. Forced air dryers can also be used to dry stalks when the number of samples is small. Stalks dried in the manner outlined above can be stored at room temperature in a lab or similar climate-controlled space with no noticeable degradation of material properties for a period of several years. Before data collection in the lab, the stalks were cleaned by snipping the remaining parts of the brace roots as close to the node as possible and peeling off any remaining parts of the leaf sheaths. Any barcode tags that had become loose were firmly secured to the respective stalks. For all lab-based data collection, the identity of the stalks was recorded by scanning the QR codes using the portable Bluetooth-enabled barcode scanner explained previously for field-based data collection.

### Major diameter measurements

Maize stalks have an elliptical cross-section and, therefore, possess a major diameter across the longer axis and a minor diameter across the shorter axis [[7], [10],18] as shown in Fig. 2. The major diameter was measured using a set of calipers with Bluetooth connectivity (Mahr, Greenville, SC) in tandem with a Google spreadsheet. At the start of a phenotyping session, the Marcom software provided with the Bluetooth calipers was opened, the barcode scanner and calipers were paired with the computer, and a Google spreadsheet was opened. The spreadsheet contained the column headers as shown in Table 1, and the cursor was set in the first open cell under the barcode column. Each stalk was scanned with the barcode scanner and the barcode scanner was configured to automatically tab over to the next adjacent column after scanning the barcode. Major diameter measurements were then taken at the midspan of every internode on the major axis starting at the ear-bearing internode followed by the subsequent basal internodes. The flats of the caliper were positioned to lie flat against the ear groove ensuring proper contact of the caliper with the stalk and avoiding excessive force to prevent deformation of the stalks resulting in inaccurate measurements. The calipers were configured so that after a value was sent to the computer, the cursor automatically tabbed over to the next adjacent cell. All measurements were taken in centimeters. After completing all measurements for a stalk, the cursor was manually moved to the next row of the spreadsheet, and the process was repeated for the next stalk.

**Table 1:** Example of spreadsheet used to collect major diameters.

Stalk ID	Major Diameter 1(mm)	Major Diameter 2(mm)	Major Diameter 3(mm)	Major Diameter 4(mm)	Major Diameter 5(mm)

### Internode length measurements

Internode lengths were calculated by acquiring calibrated images of each stalk that were analyzed using a machine-learning script written in R/Python. The algorithm automatically detects each node of the stalk from the image and uses the total length of the stalk and the location of nodes to calculate length of individual internodes. To acquire the calibrated images, a stalk was placed on a 48-inch x 36-inch grid with the bottom of the stalk being placed near the top of the grid. A Canon EOS Rebel SL3 camera with an EF-S 18–55 mm 1:4–5.6 IS STM zoom lens (Canon, Melville, NY) was positioned directly above the stalk using a light stand (LS-CT40MB, Impact, Orlando, FL). The distance between the camera lens and the stalk was set at approximately 64 inches by default. The distance was increased when needed to ensure the entire stalk fit inside of the field of view of the camera. The camera was connected to a computer wirelessly using the EOS software provided by the manufacturer. The EOS software was used to capture an image of the stalk. The file location where the image was saved was open on half of the computer screen. The file name of the image was then changed by pressing F2 (hot key for renaming a file) on the keyboard and the StalkID was scanned. The scanner was configured to append an ‘enter’ character after scanning. Thus, each file was automatically named according to the unique stalk ID (i.e., barcode). Configuring the scanner to append ‘enter’ after scanning and using the F2 hot key significantly improved throughput and reduced the chance of human error.

Following image collection, image preprocessing was performed to ensure uniformity in the orientation of input images and focus object detection within a standardized background area. All files were compiled into a single target directory, and the images were manually checked to ensure the base of each stalk was positioned at the top of individual images. Full-sized images were then cropped using a custom Python script to isolate the stalk and underlying imaging surface supporting the stalk to achieve an approximately 55% reduction in the image size.

To automate maize stalk internodal region measurements, the YOLOv5m (“YOLOv5 by Ultralytics [[22]on 7.0],” 2022) object detection algorithm was trained to identify the nodal regions of maize stalks. YOLOv5 is a single-stage object detection model able to predict the location and category of an object with broad utility, high accuracy, and rapid processing times [3,^5^,21]. Although several versions of the YOLOv5 model exist, the YOLOv5m model provides an ideal balance between neural network complexity and algorithm detection speeds. The model was re-trained to identify the nodal regions of maize stalks using a custom dataset of imaged maize stalks. The workflow to measure nodal images can be briefly described as follows. Stalk images are compiled into a single directory for analysis. Nodal inference is performed using the detect.py script supplied in the yolov5 repo before LabelImg [20] is used to manually verify nodal detections. Erroneous nodal detections are relatively rare and typically attributed to a physical stalk label covering a node, leaf sheaths crossing over the surface of a stalk being, or a blurry photo caused by someone disturbing the imaging setup. 99% of nodal annotations were correctly and accurately placed following manual verification.

Once verified, a set of custom R scripts were used to produce the final internode lengths. These functions associate image dimensions with filename identifiers, compile all verified nodal annotation data into a single data frame, calculate the Euclidian pixel distance between the midpoints of vertically sorted nodal annotations across individual stalks, convert pixel distances to physical distances using the known dimensions of the imaging background, and output internodal lengths as a single excel file of stalk/internode identifiers and lengths. All scripts, example images, and a tutorial on setup and usage are available on Github at https://github.com/nbo245/InterMeas and a Shiny dashboard can be run locally to implement the internode measurement workflow within an interactive GUI environment.

### Measurements of minor diameter, rind thickness, rind penetration resistance and integrated puncture score

**Fig. 1:**
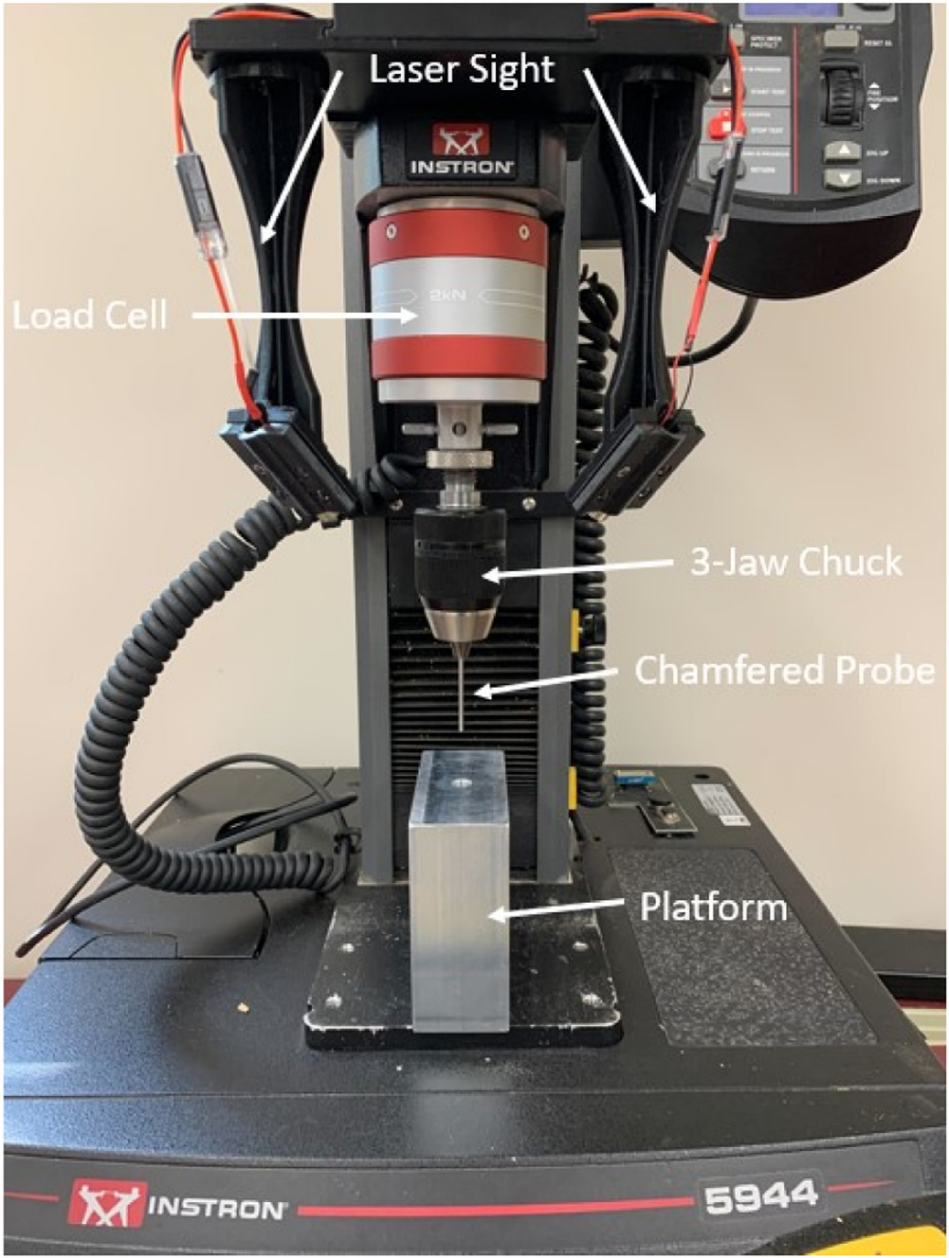
Aluminum platform, chamfered probe, 3-jaw chuck, load cell, and laser sight configuration used during rind puncture tests.

A rind puncture methodology was employed to determine the minor diameter, rind thickness, rind penetration resistance and integrated puncture score of each internode section of each stalk sample. Each internode was punctured using an Instron universal testing system (model 5940) equipped with a 2 kN load cell (Instron, Norwood, MA). The stalks were individually placed on a flat aluminum platform, that measured 1.75 inches (4.445 cm) wide, 4 (10.16 cm) inches deep, and 4 inches (10.16 cm) high. There was a hole of 0.375 inches (0.9525 cm) in diameter located in the center of the platform to allow for the puncture probe to pass completely through the stalk without impacting the platform (Fig. 1).

**Fig. 2:**
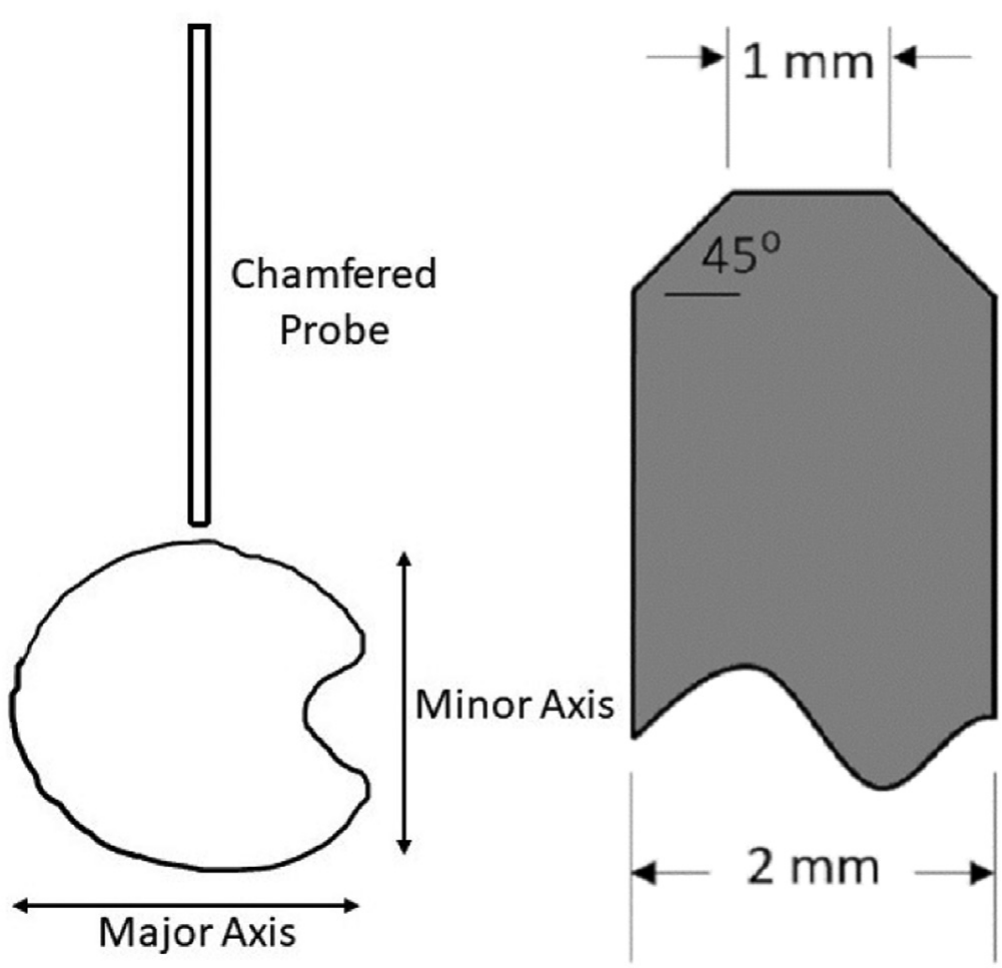
Cross-section of cornstalk showing where the stalk should be punctured during RPR and the chamfered probe geometry.

**Fig. 3:**
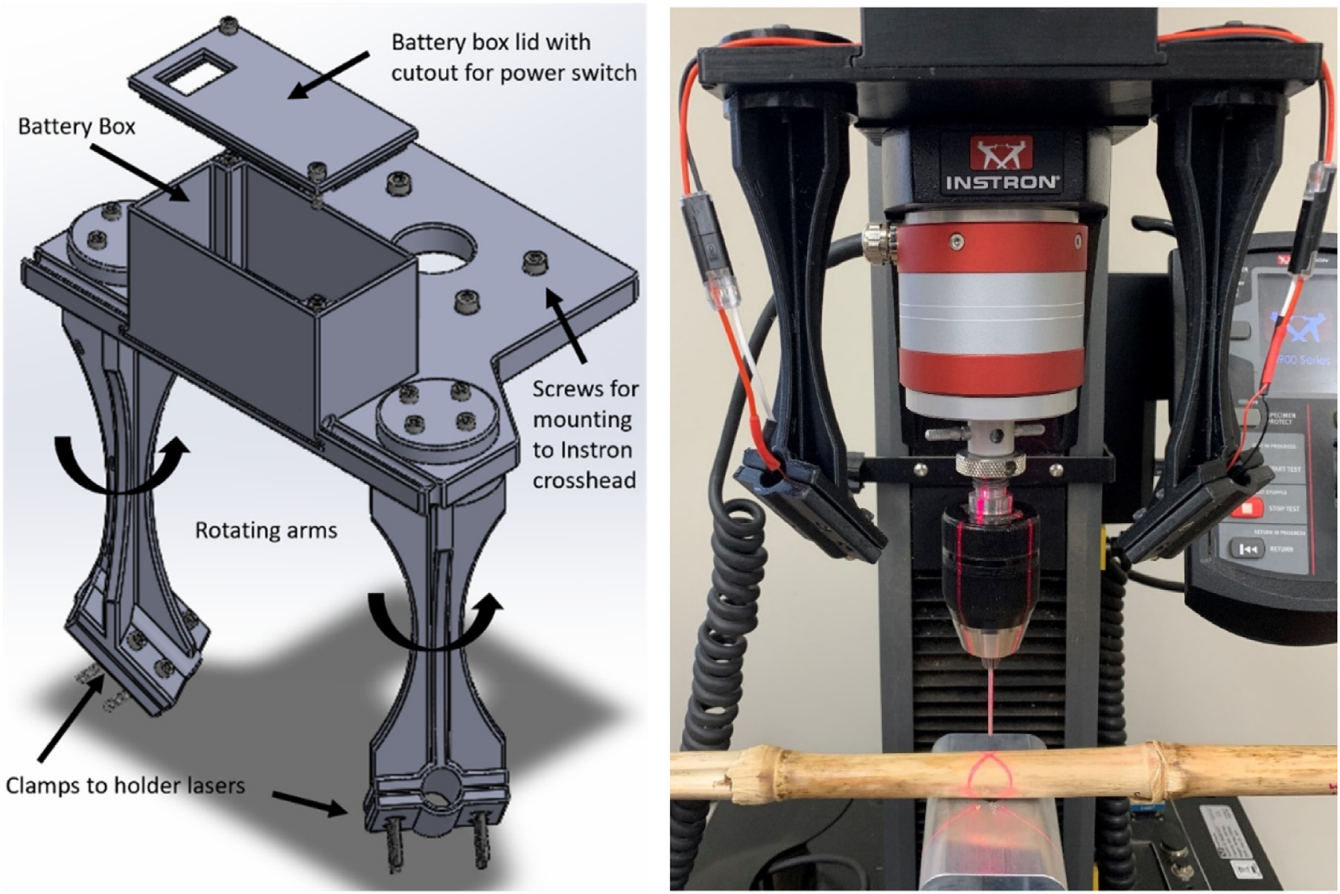
3D printed laser sight to increase accuracy and throughput of rind penetration tests.

The rind puncture probe was 6.25 centimeter long, 2 mm in diameter with a 45-degree chamfer that reduced the diameter at the tip of the probe to 1 mm [8]. The probe was affixed to the moving head of the Instron test frame using a 3-jaw chuck. The chuck gripped the top 2.5 cm of the probe leaving approximately 3.75 cm of the probe extending beyond the mouth of the 3-jaw chuck. The tip of the probe was zeroed against the face of the flat aluminum platform such that the Instron measured zero displacement when the tip of the probe was in line with the top surface of the aluminum platform. Before puncturing the stalk, the probe was raised 35 mm above zero and then displaced at a rate of 25.4 mm /second until the tip of the probe extended 5 mm below the platform surface. The ear-bearing internode of each stalk was punctured first followed by the more basal internodes. This enabled the Instron software to automatically record the internode number of each test. Each internode was punctured in the center of the internode through the minor axis of the stalk (Fig. 2). The force displacement To facilitate aligning the center of the stalk with the puncture probe, a custom 3D-printed laser sight was mounted on the movable head of the Instron (Fig. 3). The arms of the laser sight were aligned such that two laser lines intersected at the point on the stalk which would be punctured by the probe. This setup is similar to that used on many commercially available drill presses. When calibrating and aligning the laser sights, care was taken to focus the laser by rotating the lens, and the body of the laser was also rotated to align the plane of the laser line to the puncture probe.

**Fig. 4:**
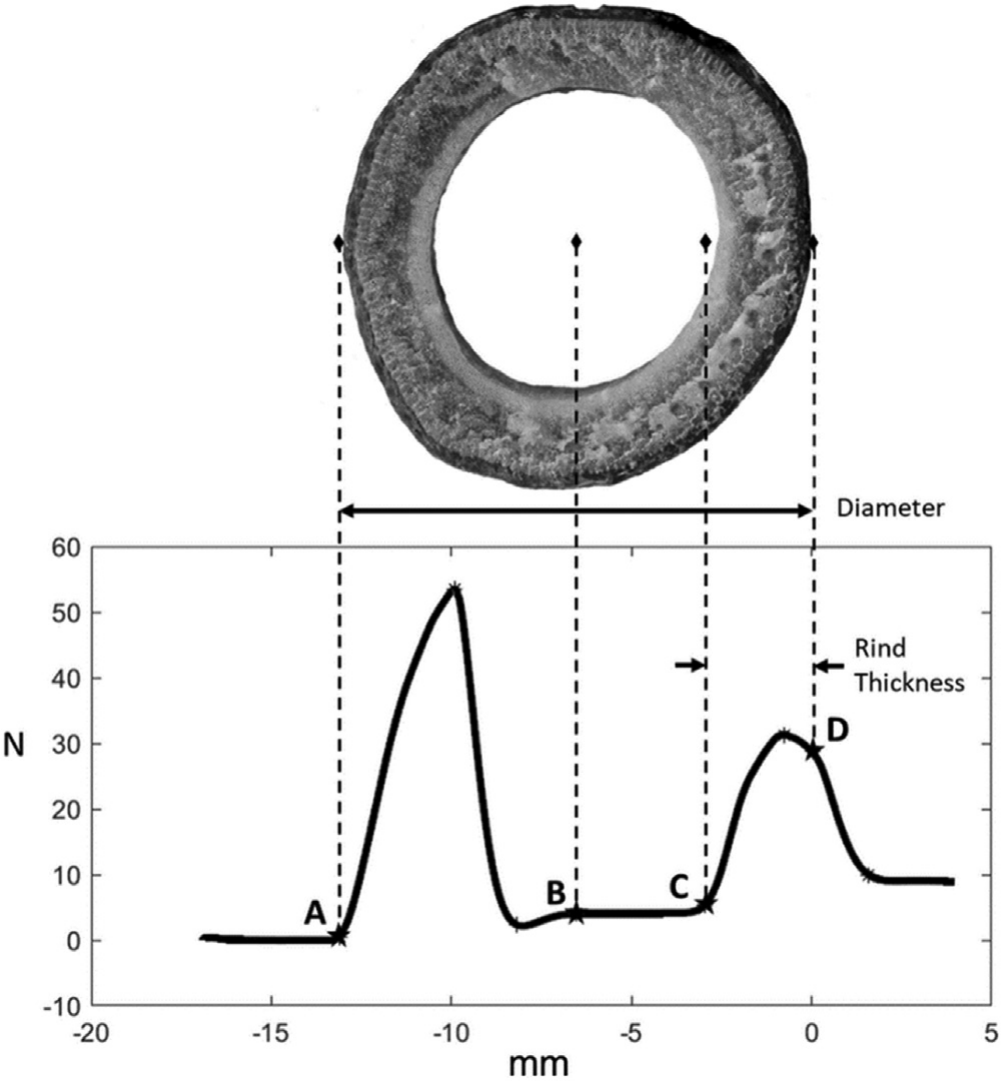
Key points of the load–extension curve from a puncture test showing how each point relates to the physical features of the stalk cross section. Labeled points are: A—Point of initial contact, B—Midpoint, C—Point of reengagement, D—Exit (zero) plane. Diameter is calculated as the distance between points A and D whereas the rind thickness is calculated as the distance between points C and D. The rind puncture resistance is the maximum force encountered during the test (adapted from [13]).

If an internode was damaged or otherwise unable to be tested, a blank test was run to enable the Instron software to keep an accurate record of the internode number being tested. These blank test files were later identified and removed from further analysis. After testing, a csv file for each internode was saved that includes time (seconds), displacement (millimeters), and force (newtons) data collected at 1000 hertz. The methods file used to run the Instron software is available as supplementary material. These files were then analyzed using an integrative puncture score algorithm [13,17] which calculated minor diameter, integrated puncture score, rind thickness, and maximum puncture force (also known as rind penetration resistance). Fig. 4 shows briefly illustrates how the force displacement data was used to calculate these quantities.

### Lab based structural bending strength and structural bending stiffness measurements

The DARLING device is commonly used to obtain in-field measurements of structural bending strength and structural bending stiffness. However, laboratory-based 3-point bending tests can also be used to obtain these measurements [11]. An advantage of lab-based tests is that the stalks have been dried and stabilized and, therefore, the testing can take place over a longer period of time. Lab-based 3-point-bending tests do not produce the same stress distribution as naturally loaded stalks but, if conducted properly, they do produce similar failure patterns observed in field-based tests. This bias in lab-based methods should be considered in the subsequent interpretations of the data. In particular, the user artificially selects the location of failure in lab-based 3-point bending test as the failure almost always occurs in the immediate vicinity of the loading anvil. In contrast, field-based methods which utilize a DARLING device allow the stalk to fail at the location that is structurally the weakest (i.e., the failure location is not artificially selected by the user but determined by the unique geometry and material properties of the stalk). It must also be mentioned that 3-point bending tests are often performed incorrectly by using short spans that produce unrealistic loadings and failure patterns [6]. For this reason, short span tests should always be avoided. Long span test in which the stalk is loaded and supported at nodes and the span is at least 10x the diameter of the stalk have been shown to produce the most natural stalk failure modes whereas short span tests produce unnatural failure patterns [9,^12^,15].

**Fig. 5:**
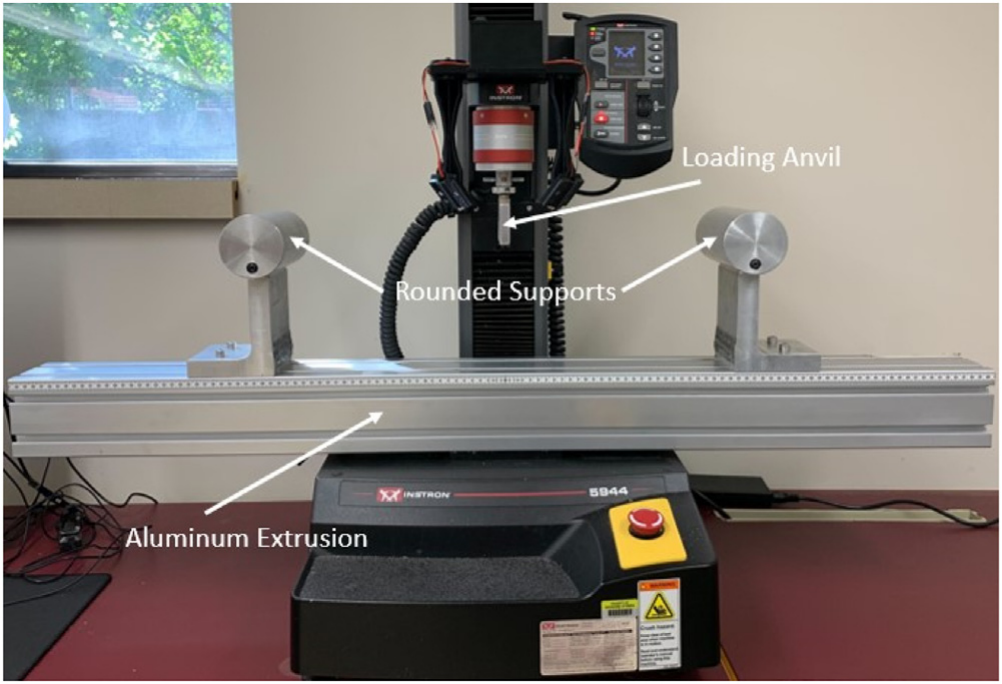
Three-point-bending fixture that includes loading anvil, an aluminum extrusion, and rounded supports.

**Fig. 6:**
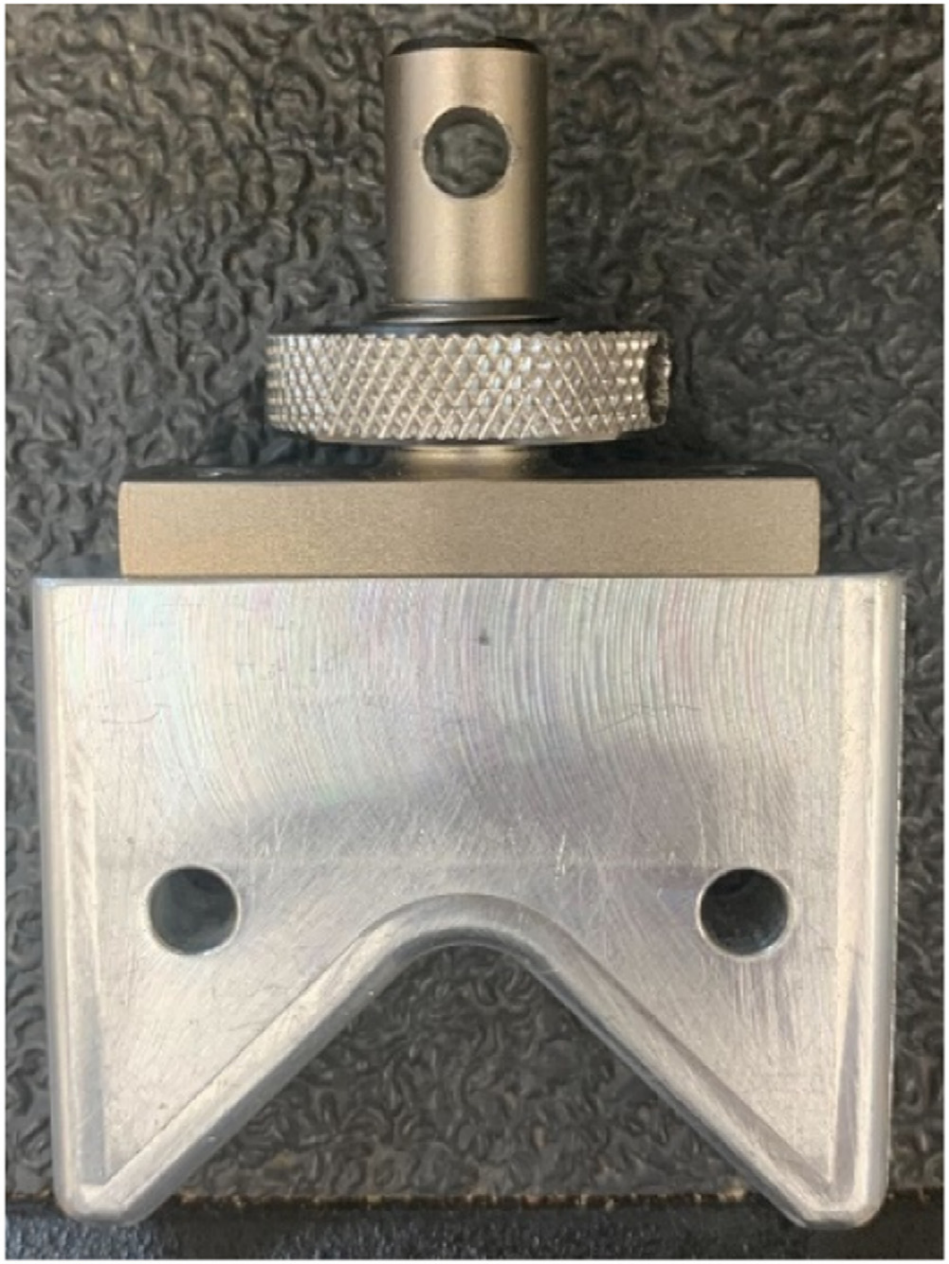
Inverted “V” loading anvil that allows for more contact than a flat surface and prevents the cross section from being crushed.

To perform long span 3-pt bending test a custom fixture was mounted to a universal testing system. The base of the fixture was a 4-inch x 4-inch x 48-inch aluminum extrusion that had two l-shaped brackets approximately 6 inches tall affixed with 3-inch cylinders to provide a round surface for placing the stalks (Fig. 5). A custom anvil with a large radius was used to prevent stress concentrations. The anvil shape (Fig. 6) is an inverted “V” to allow for more contact than a flat surface thereby reducing the chance of the cross section being crushed in compression during the test. The stalks were loaded at the most central node and supported at the most apical and basal nodes. The 3-point bending tests were run at 10 centimeter-minute^–1^ displacement rate, and data was collected every 100 milliseconds. If the stalk rolled during testing or did not remain stably affixed to the supports, then the test data was discarded. It should be noted that due to irregular geometries some stalks cannot be accurately in 3-pt bending as they will tend to twist or roll during the test. Force displacement data from each test was used to calculate the induced bending moment (*M*) using the following equations for non-symmetric three-point bending from [4].

(4) $$M\left(x\right)=\frac{Fbx}{L},\;0\leq x\leq a$$

(5) $$M\left(x\right)=\frac{Fa}{L}\left(L-x\right),a<x\leq L$$

where *F* is the applied load, *x* is the distance along the stalk measured from its left side, *a* and *b* are the distances to the applied load measured from the supports on the left and right sides of the stalk respectively, and *L* is the distance between supports. Bending strength was defined as the maximum moment applied to the stalk at the location of failure.

Stalk flexural stiffness, *EI*, was calculated using the following equation also from [4]:

(6) $$EI=\;\frac{\emptyset a^{2}b^{2}}{3L}$$

Where ∅ is the slope of the force-displacement curve, acquired by flexing the stalk in three-point bending by less than 6 mm , *a* and *b* are the distances measured from the supports on the left and right sides of the stalk respectively and *L* is the distance between the supports. This small deflection of less than 6 mm did not introduce any permanent damage to the stalk.

### Calculation of linear density

The linear density of each internode section of the stalk was calculated as follows. First the stalks were cut into internodal sections using a trim saw (Hi-Tech Diamond 6″ Trim Saw). The sectioned portion of the stalk contained only internodal tissue. All tissues in and around the node (approximately 2 cm on either side of the node line) were removed. Then the sectioned length was measured in centimeters using a ruler taped to a desk. The sectioned mass was then measured in grams using a digital kitchen scale. Linear density (grams/centimeter) was then calculated using:

(7) $$Linear\;Density=\;\frac{m}{L}$$

where *m* is the sectioned mass and *L* is the sectioned length.

### Material bending strength and Youngs modulus measurements

Micro-three-point bending tests were conducted on excised sections of rind tissue from internode sections of maize stalks. Prior to testing, the stalks were cut into internodal sections using a trim saw (Hi-Tech Diamond 6″ Trim Saw). Strips of rind tissue were cut from each internode section using a razor blade. Due to tissue heterogeneity and challenges associated with material testing of small specimens a total of ten rind tissue strips were tested per internode, with each strip measuring 18-mm s x 2.8-mm s x 0.7-mm s (length x width x thickness).

The micro-three-point bending tests were performed using an Instron universal testing machine (model 5940) equipped with a 50-newton load cell and custom loading and support anvils. The support anvils had a span length of 10 mm , and the Instron software (Bluehill Universal) was used to control the testing process and collect data. To begin the testing process, a preload force of 0.001 N was initially applied to each sample to bring the testing anvil into contact with the test sample. Three preloading cycles were then applied, where the maximum engineering strain in the sample cycled between 0.3% to 1.4%. After preloading cycles were complete the sample was tested to failure. During the test, the time (seconds), displacement (millimeters), and force (newtons) values were recorded for each sample. Using equation 8 and 9, flexural stress and strain were then calculated using the span length (L), sample-cross section (width (w) and thickness (t)), and raw force-displacement (*F*, δ) data collected during the test. Using the resulting stress-strain curve, values for flexure stress at failure (σ), strain at failure (ε), and Young's modulus (*E*) were derived.

(8) $$\sigma =\frac{3FL}{2wt^{2}}$$

(9) $$\varepsilon =\frac{6\delta }{wt^{2}}$$

(10) $$E=\frac{\sigma }{\varepsilon }$$

### Chemical composition

**Fig. 7:**
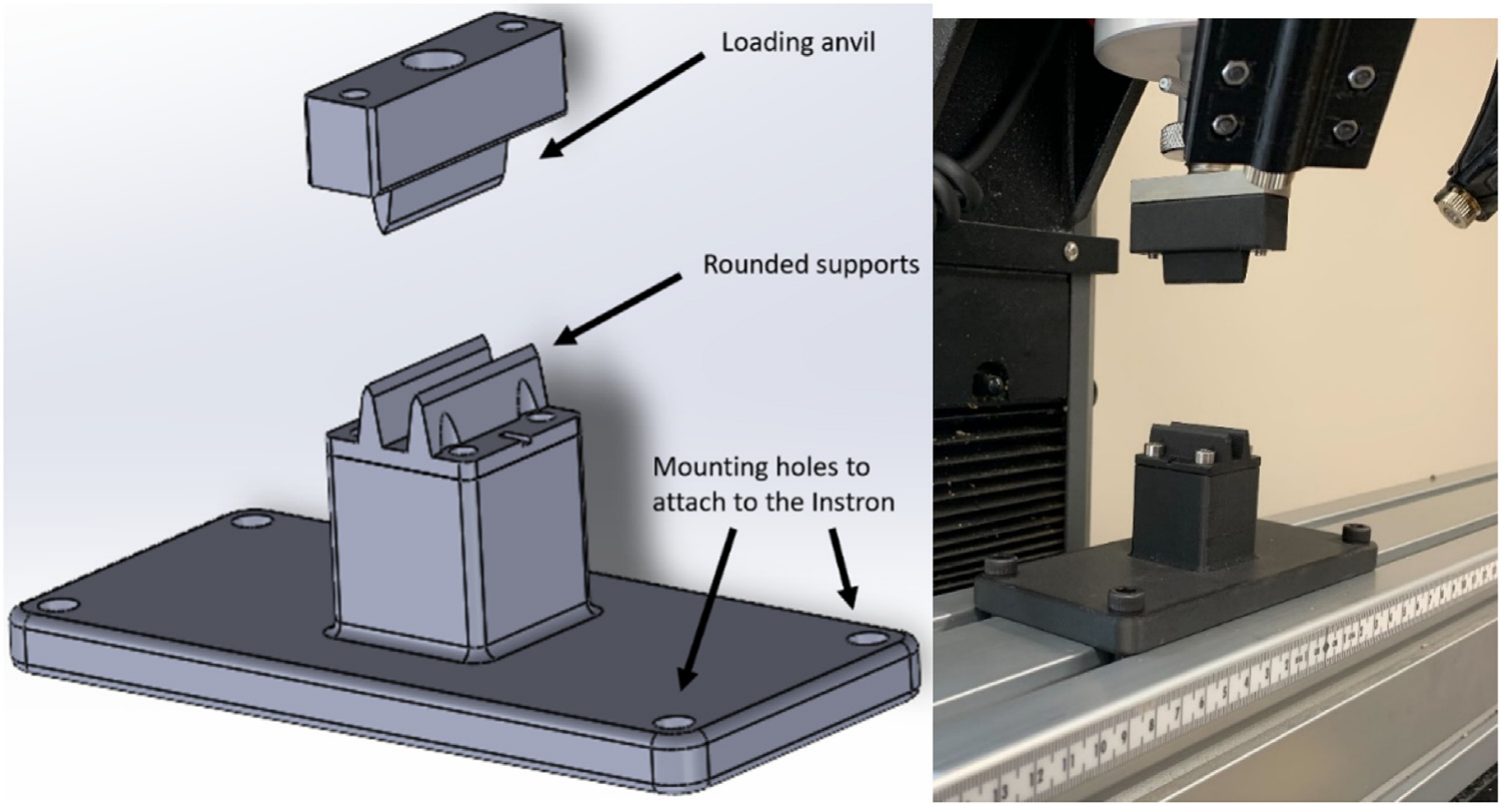
Micro-3-pt Bending fixture.

To study the biochemical composition of stalks in relation to stalk lodging, the structural carbohydrates in the stalk were quantified, including cellulose, hemicellulose, as well as a secondary metabolite, lignin. To quantify these biomolecules, stalk samples from each genotype were pooled. Particularly, six stalks per plot (three stalks from each row) were randomly sampled per genotype per replication. The internode immediately below the primary ear-bearing node (ear internode) and the lower-most elongated internode (bottom internode) of collected stalks were excised from the stalks and ground separately in a Retsch SM 300 cutting mill at 1700 rpm. The ground biomass was sifted using a 2 mm sieve and the material recovered was further hand ground with a pestle and mortar using liquid nitrogen to increase the particle fineness. Within each replication, these ground samples were pooled to generate the respective internode-wise samples per genotype. For wet lab procedures, 5 gs of sample per genotype per replication was sent to Dairyland Laboratories, Inc. (Arcadia, WI). Acid detergent fibers (ADF), ash-free Neutral detergent fiber (aNDF), and lignin proportions were measured using the standardized AOAC official methods. Cellulose and hemicellulose values were obtained by calculating the difference between ADF and lignin proportions and aNDF and ADF proportions of samples, respectively. Fig. 7

## Ethics statements

No ethic statements to declare.

## University of Idaho

Lucas Debilius, Anthony DeSantis, Jessy Faulkner, Zane Holiday, Ethan Morris, Nathan LaVoie, Juhyung Lee, Serena Strawn and Andrew Stucker

**Clemson University:** William Betsill, Rebecca Bishop, Benjamin Clark, Meredith Cobb, Andra Cummings, Bryce Deuty, Grace Gaston, Emma Hatchell, Benjamin Herron, Daniel Hiott, Kaila Honakar, Alexandria Jajack, Rikki Johnson, Grant Kroeschell, Clare Mazzeo, Georgia Moore, Jonathan Tan, Venkata Tatineni and Alison Wuerfel.

**University of Kentucky:** Howard Gates, Abigail Haley, Osei Jordan, Jordan Luciano, Nick Rydz, Zoe Schroeder, Ilya Segal, Evanson Telisme, Joseph Woomer, Ren Young, and Jingxia Zhong

## CRediT authorship contribution statement

**Kaitlin Tabaracci:** Writing – original draft, Writing – review & editing, Investigation, Methodology, Data curation. **Norbert T. Bokros:** Software, Investigation, Data curation, Writing – review & editing, Visualization, Methodology. **Yusuf Oduntan:** Investigation, Methodology. **Bharath Kunduru:** Investigation, Methodology, Writing – review & editing. **Joseph DeKold:** Investigation, Methodology. **Endalkachew Mengistie:** Investigation, Methodology. **Armando McDonald:** Resources, Funding acquisition, Methodology. **Christopher J. Stubbs:** Investigation, Methodology, Writing – review & editing. **Rajandeep S. Sekhon:** Resources, Funding acquisition, Project administration, Supervision, Conceptualization, Writing – review & editing. **Seth DeBolt:** Resources, Funding acquisition, Project administration, Conceptualization, Supervision. **Daniel J. Robertson:** Resources, Funding acquisition, Project administration, Conceptualization, Supervision, Investigation, Methodology, Data curation, Writing – original draft, Writing – review & editing.

## Declaration of competing interest

The authors declare that they have no known competing financial interests or personal relationships that could have appeared to influence the work reported in this paper.

## Data Availability

Data will be made available on request.

## Appendix Supplementary materials

Image, application 1
