# Supplementary material for: Biomechanical phenotyping pipeline for stalk lodging resistance in maize
Source: MethodsX. 2024 Jan 9;12:102562. doi: 10.1016/j.mex.2024.102562 (PMC10825676; doi:10.1016/j.mex.2024.102562)
Supplement: Supplementary file 1 [file mmc1.zip › Supplimentary Material/3-pt Bending/Manufacturing Plans/Solidworks Files & Drawings/Base/3-pt Bending Base.PDF]

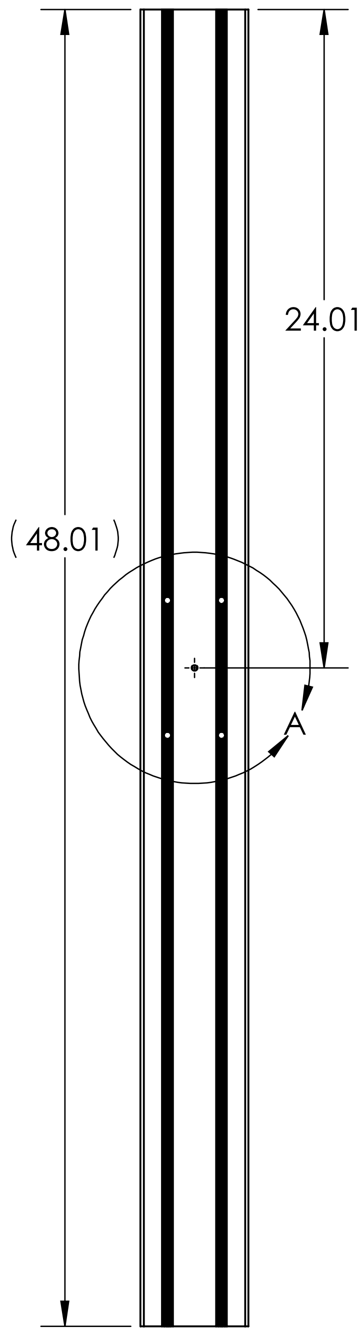

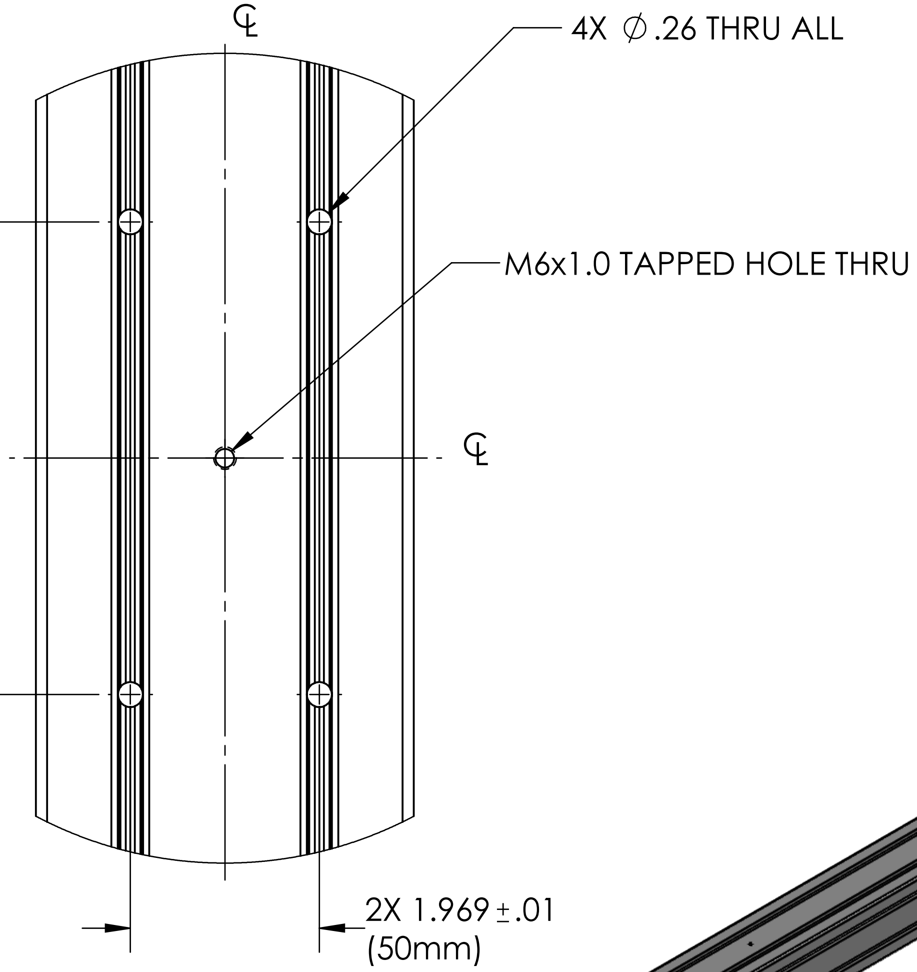

DETAIL A
SCALE 1 : 2

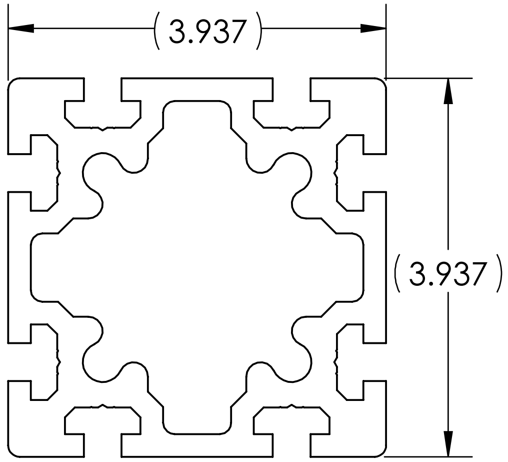

SCALE 1 : 2

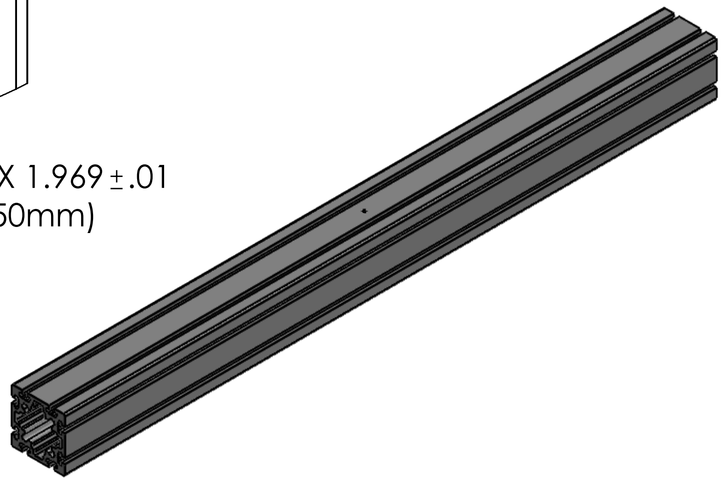

**PROPRIETARY AND CONFIDENTIAL** THE INFORMATION CONTAINED IN THIS DRAWING IS THE SOLE PROPERTY OF UNIVERSITY OF IDAHO, ME DEPARTMENT. ANY REPRODUCTION IN PART OR AS A WHOLE WITHOUT THE WRITTEN PERMISSION OF UNIVERSITY OF IDAHO, ME DEPARTMENT IS PROHIBITED.	DIMENSIONS ARE IN INCHES<br>THIRD ANGLE PROJECTION<br>MATERIAL: ALUMINUM		3 POINT BEND FIXTURE	
DEFAULT TOLERANCES:	DESCRIPTION: BASE BEAM		**UNIVERSITY OF IDAHO ME DEPARTMENT**	
LINEAR: X. ±.25 X.X±.1 X.XX±.01 X.XXX±.002 / ANGULAR: X. ± 2 X.X± 1 X.XX± 0 30'	CHECKED BY: XXXXXXXXXX	DATE: XX/XX/XX		
	DRAWN BY: TAYLOR SPENCE	DATE: 4/25/2019	PART #: -	QTY: 1
	FILE NAME: Base Modified V2.SLDPRT		SCALE: 1:10	SHEET: 1 OF 1
