# Supplementary material for: Biomechanical phenotyping pipeline for stalk lodging resistance in maize
Source: MethodsX. 2024 Jan 9;12:102562. doi: 10.1016/j.mex.2024.102562 (PMC10825676; doi:10.1016/j.mex.2024.102562)
Supplement: Supplementary file 1 [file mmc1.zip › Supplimentary Material/3-pt Bending/Manufacturing Plans/Solidworks Files & Drawings/Bending Anvil/3-pt Bending Anvil.PDF]

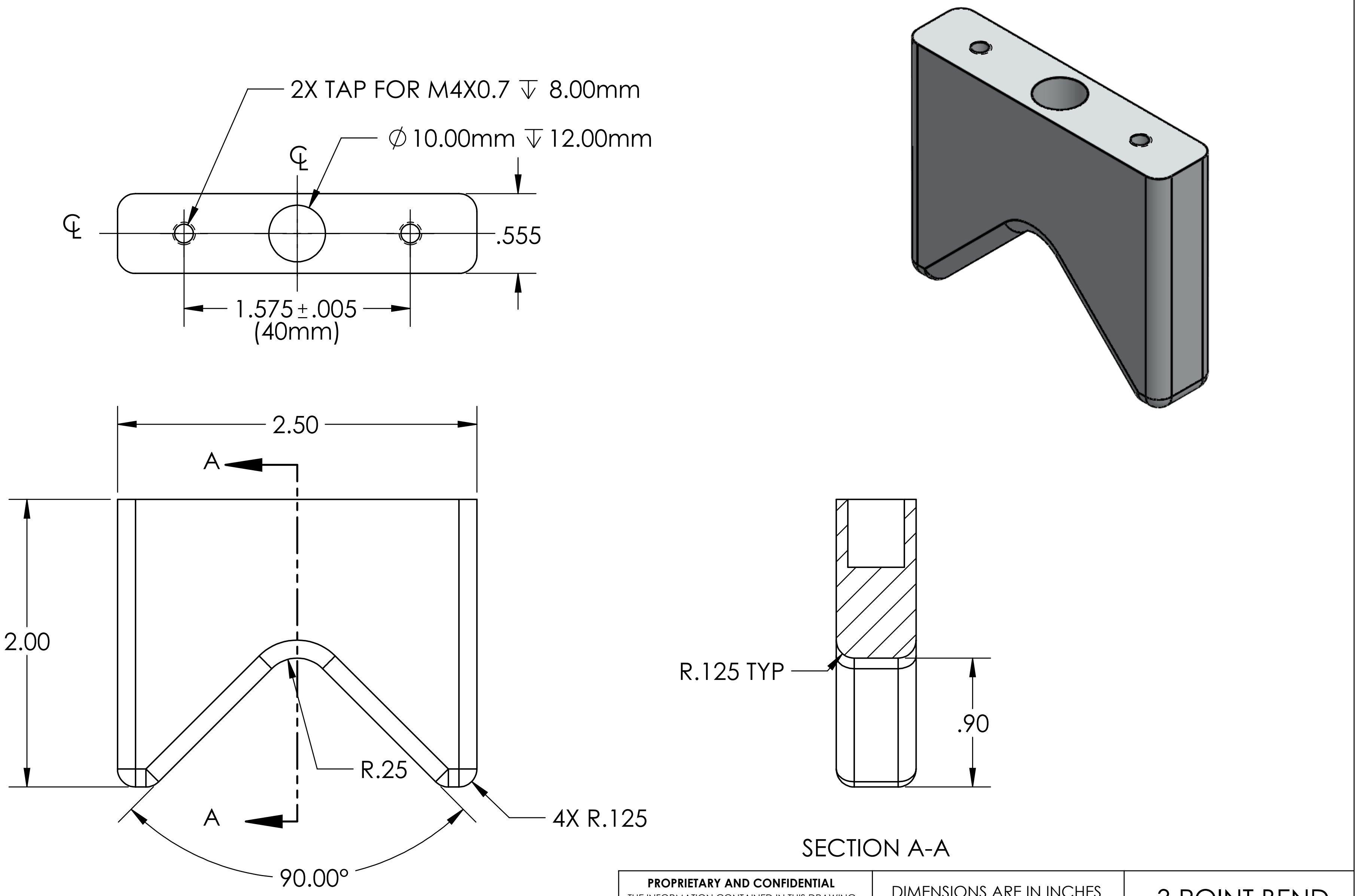


| | | |
|---|---|---|
| **PROPRIETARY AND CONFIDENTIAL**<br>THE INFORMATION CONTAINED IN THIS DRAWING IS THE SOLE PROPERTY OF UNIVERSITY OF IDAHO, ME DEPARTMENT. ANY REPRODUCTION IN PART OR AS A WHOLE WITHOUT THE WRITTEN PERMISSION OF UNIVERSITY OF IDAHO, ME DEPARTMENT IS PROHIBITED. | DIMENSIONS ARE IN INCHES<br>THIRD ANGLE PROJECTION<br>MATERIAL: ALUMINUM | 3 POINT BEND FIXTURE |

| | | | | | |
|---|---|---|---|---|---|
| DEFAULT TOLERANCES: | | DESCRIPTION: BENDING HEAD | | **UNIVERSITY OF IDAHO ME DEPARTMENT** | |
| LINEAR:<br>X.±.25<br>X.X±.1<br>X.XX±.01<br>X.XXX±.002 | ANGULAR:<br>X.±2<br>X.X±1<br>X.XX±0 30' | CHECKED BY: XXXXXXXXXX | DATE: XX/XX/XX | | |
| | | DRAWN BY: TAYLOR SPENCE | DATE: 4/11/2019 | PART #: | QTY: |
| | | FILE NAME: Bending Head Final.SLDPRT | | SCALE: 1:1 | SHEET: 1 OF 1 |
