# Supplementary material for: Biomechanical phenotyping pipeline for stalk lodging resistance in maize
Source: MethodsX. 2024 Jan 9;12:102562. doi: 10.1016/j.mex.2024.102562 (PMC10825676; doi:10.1016/j.mex.2024.102562)
Supplement: Supplementary file 1 [file mmc1.zip › Supplimentary Material/3-pt Bending/Manufacturing Plans/Solidworks Files & Drawings/Supports/Small Support Head.PDF]

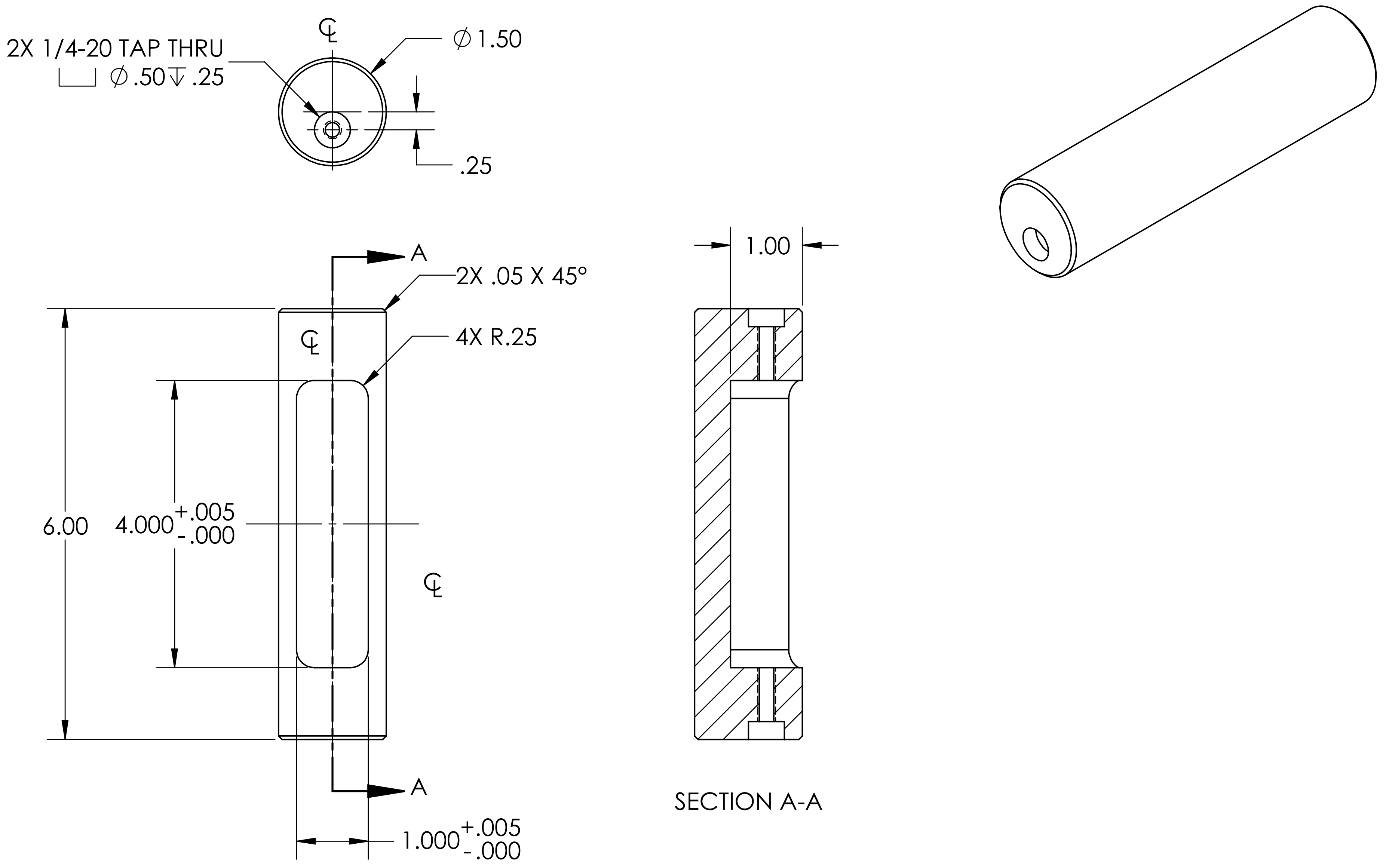

PROPRIETARY AND CONFIDENTIAL

THE INFORMATION CONTAINED IN THIS DRAWING IS THE SOLE PROPERTY OF UNIVERSITY OF IDAHO, ME DEPARTMENT. ANY REPRODUCTION IN PART OR AS A WHOLE WITHOUT THE WRITTEN PERMISSION OF UNIVERSITY OF IDAHO, ME DEPARTMENT IS PROHIBITED.

DIMENSIONS ARE IN INCHES
THIRD ANGLE PROJECTION

MATERIAL: ALUMINIUM

3 POINT BEND FIXTURE

DEFAULT TOLERANCES:

LINEAR:	ANGULAR:
X. ±.25	X. ± 2
X.X±.1	X.X± 1
X.XX±.01	X.XX± 0 30'
X.XXX±.002	

DESCRIPTION: SMALL SUPPORT HEAD

CHECKED BY: XXXXXXXXXX DATE: XX/XX/XX

DRAWN BY: TAYLOR SPENCE DATE: 4/11/2019

FILE NAME: Small Support Head V3.SLDPRT

UNIVERSITY OF IDAHO ME DEPARTMENT

PART #: -
QTY:
SCALE: 1:2
SHEET: 1 OF 1
