# Supplementary material for: Biomechanical phenotyping pipeline for stalk lodging resistance in maize
Source: MethodsX. 2024 Jan 9;12:102562. doi: 10.1016/j.mex.2024.102562 (PMC10825676; doi:10.1016/j.mex.2024.102562)
Supplement: Supplementary file 1 [file mmc1.zip › Supplimentary Material/3-pt Bending/Manufacturing Plans/Solidworks Files & Drawings/Supports/Support Head.PDF]

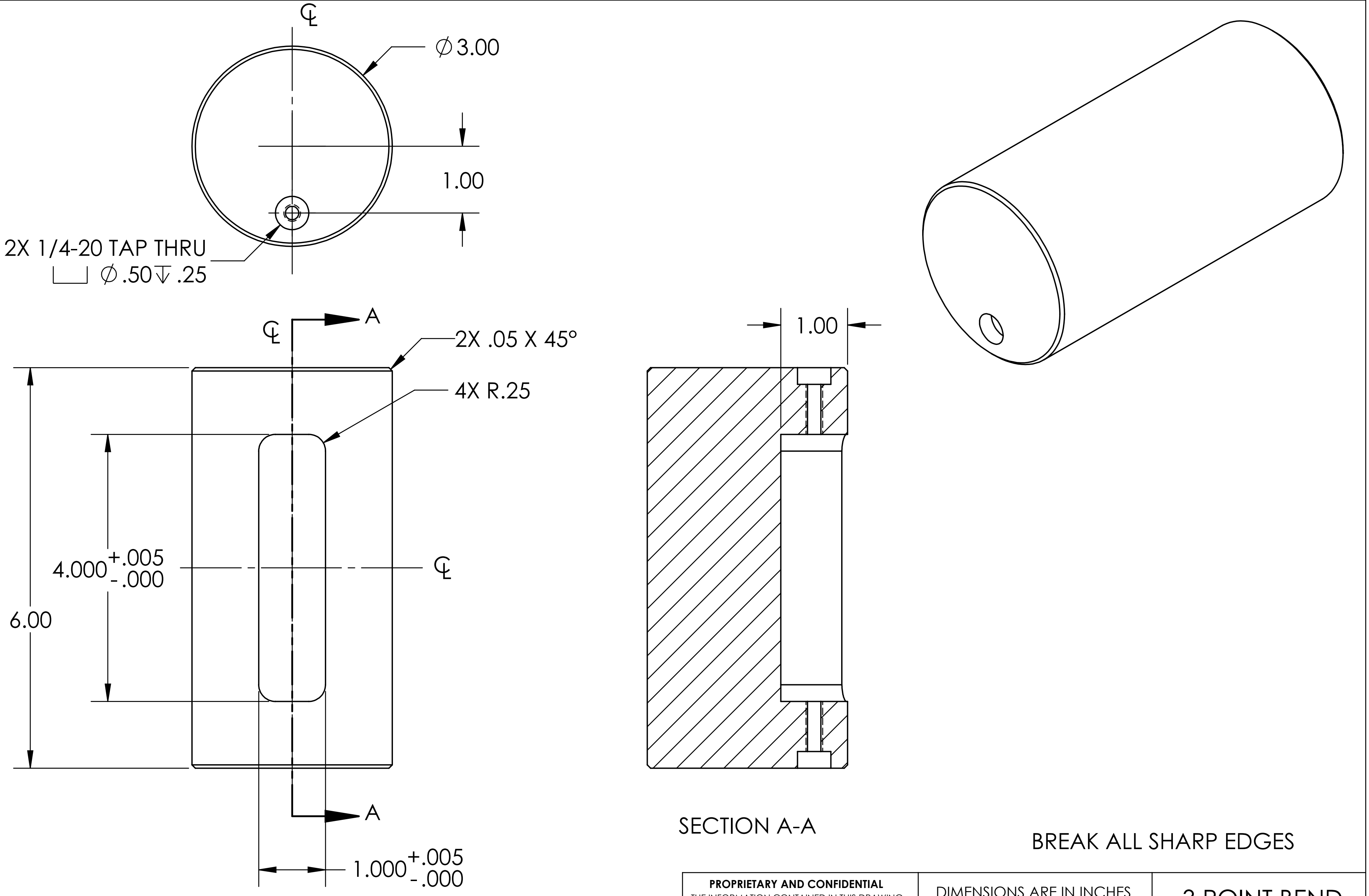

BREAK ALL SHARP EDGES

PROPRIETARY AND CONFIDENTIAL

THE INFORMATION CONTAINED IN THIS DRAWING IS THE SOLE PROPERTY OF UNIVERSITY OF IDAHO, ME DEPARTMENT. ANY REPRODUCTION IN PART OR AS A WHOLE WITHOUT THE WRITTEN PERMISSION OF UNIVERSITY OF IDAHO, ME DEPARTMENT IS PROHIBITED.

DIMENSIONS ARE IN INCHES
THIRD ANGLE PROJECTION

MATERIAL: ALUMINIUM

3 POINT BEND FIXTURE

DEFAULT TOLERANCES:

LINEAR:	ANGULAR:
X. ±.25	X. ± 2
X.X ±.1	X.X ± 1
X.XX ±.01	X.XX ± 0 30'
X.XXX ±.002	

DESCRIPTION: SUPPORT LEG HEAD

CHECKED BY: XXXXXXXXXX DATE: XX/XX/XX

DRAWN BY: TAYLOR SPENCE DATE: 4/4/2019

FILE NAME: Support Leg Head V3.SLDPRT

UNIVERSITY OF IDAHO
ME DEPARTMENT

PART #: - QTY:

SCALE: 1:2 SHEET: 1 OF 1
