# Supplementary material for: Biomechanical phenotyping pipeline for stalk lodging resistance in maize
Source: MethodsX. 2024 Jan 9;12:102562. doi: 10.1016/j.mex.2024.102562 (PMC10825676; doi:10.1016/j.mex.2024.102562)
Supplement: Supplementary file 1 [file mmc1.zip › Supplimentary Material/RPR/Manufacturing Plans/RPR Chuck/Arbor.pdf]

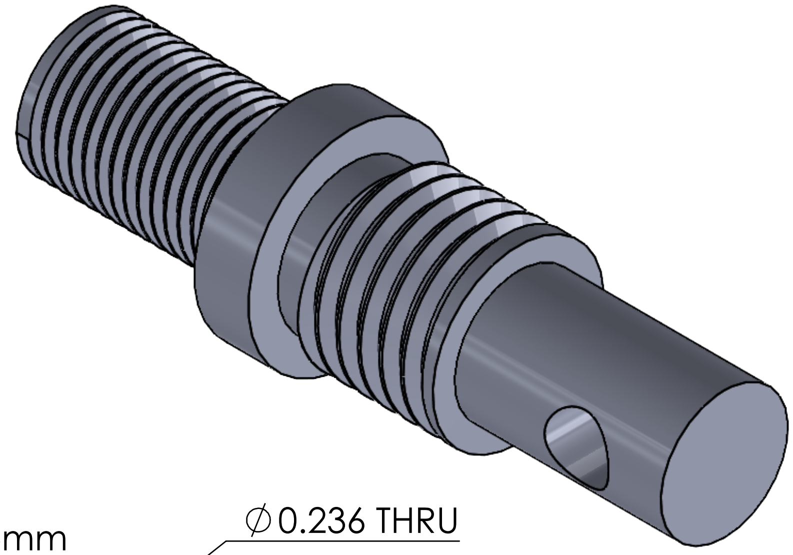

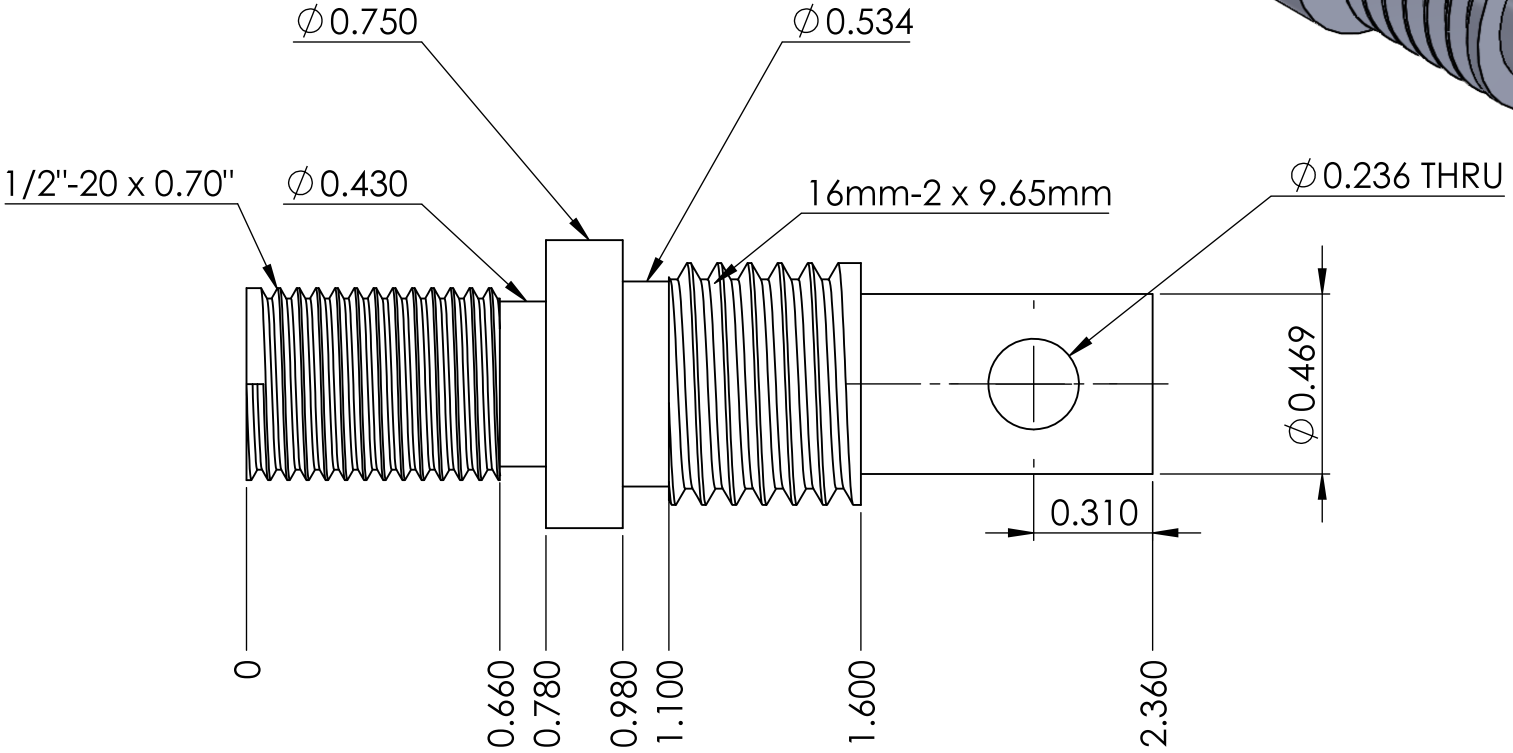

PROPRIETARY AND CONFIDENTIAL

THE INFORMATION CONTAINED IN THIS DRAWING IS THE SOLE PROPERTY OF UNIVERSITY OF IDAHO, ME DEPARTMENT. ANY REPRODUCTION IN PART OR AS A WHOLE WITHOUT THE WRITTEN PERMISSION OF UNIVERSITY OF IDAHO, ME DEPARTMENT IS PROHIBITED.

DIMENSIONS ARE IN INCHES
THIRD ANGLE PROJECTION

PROJECT TITLE:
Arbor

DEFAULT TOLERANCES:

LINEAR:	ANGULAR:
X. ±.25	X. ± 2
X.X ±.1	X.X ± 1
X.XX ±.01	X.XX ± 0 30'
X.XXX ±.002	

DESCRIPTION:

FILE NAME/PART NUMBER: Arbor

UNIVERSITY OF IDAHO
ME DEPARTMENT

SHEET: 1 OF 2
REVISION:
SCALE: 2:1

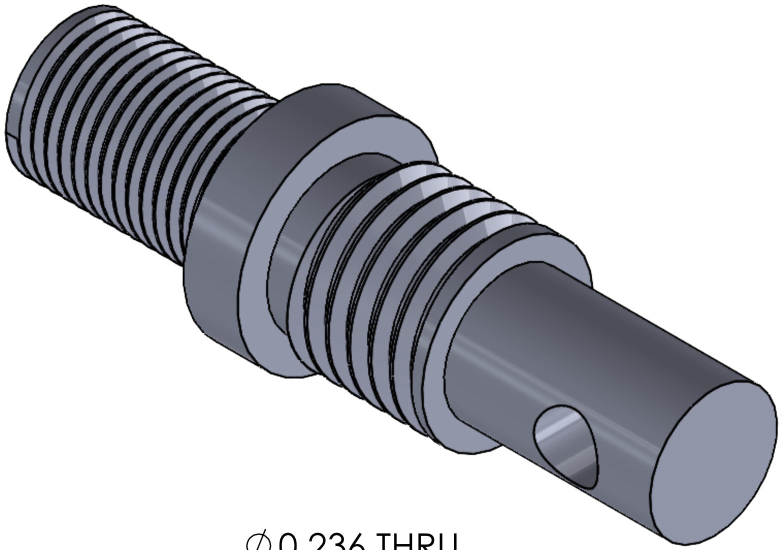

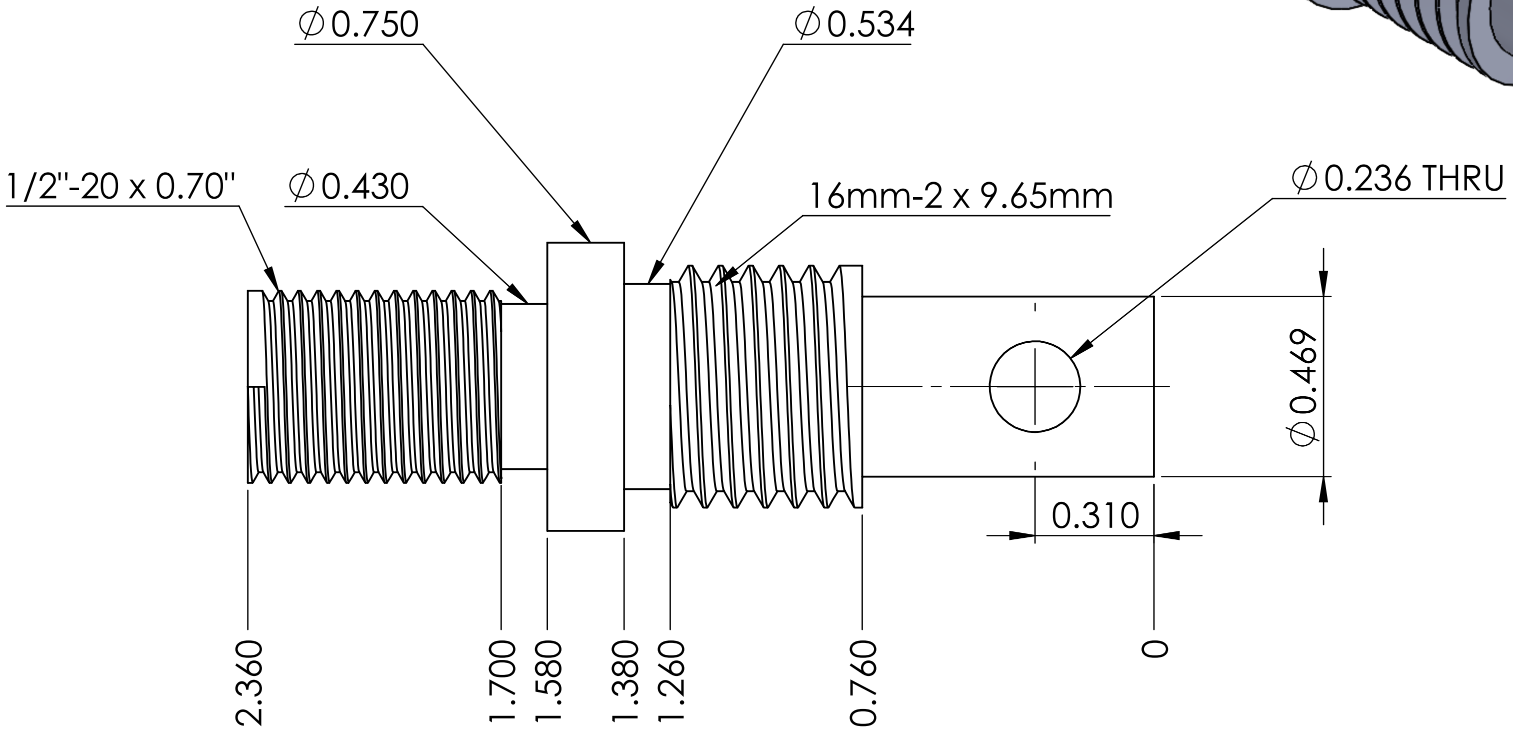

PROPRIETARY AND CONFIDENTIAL

THE INFORMATION CONTAINED IN THIS DRAWING IS THE SOLE PROPERTY OF UNIVERSITY OF IDAHO, ME DEPARTMENT. ANY REPRODUCTION IN PART OR AS A WHOLE WITHOUT THE WRITTEN PERMISSION OF UNIVERSITY OF IDAHO, ME DEPARTMENT IS PROHIBITED.

DIMENSIONS ARE IN INCHES
THIRD ANGLE PROJECTION

PROJECT TITLE:
Project Title

DEFAULT TOLERANCES:

LINEAR:	ANGULAR:
X. ±.25	X. ± 2
X.X ±.1	X.X ± 1
X.XX ±.01	X.XX ± 0 30'
X.XXX ±.002	

DESCRIPTION:

FILE NAME/PART NUMBER: Arbor

UNIVERSITY OF IDAHO
ME DEPARTMENT

SHEET: 2 OF 2
REVISION:
SCALE: 2:1
