# Supplementary material for: Biomechanical phenotyping pipeline for stalk lodging resistance in maize
Source: MethodsX. 2024 Jan 9;12:102562. doi: 10.1016/j.mex.2024.102562 (PMC10825676; doi:10.1016/j.mex.2024.102562)
Supplement: Supplementary file 1 [file mmc1.zip › Supplimentary Material/RPR/Manufacturing Plans/RPR Needles/Material for Needles.docx]

Material for Needles

<https://www.mcmaster.com/3180T31/>

Note:

Needles are 2.5” long.

4 Needles are possible from 1 ft.
