# Supplementary material for: Biomechanical phenotyping pipeline for stalk lodging resistance in maize
Source: MethodsX. 2024 Jan 9;12:102562. doi: 10.1016/j.mex.2024.102562 (PMC10825676; doi:10.1016/j.mex.2024.102562)
Supplement: Supplementary file 1 [file mmc1.zip › Supplimentary Material/RPR/Manufacturing Plans/RPR Needles/Puncture Needle.pdf]

CONSISTENCY BETWEEN THE NEEDLE'S HEAD GEOMETRY THROUGHOUT THE BATCH IS GENERALLY MORE IMPORTANT THAN THE EXACTNESS OF THE CHAMFER ON THE HEAD (SINCE WE HAVE NO ACCURATE WAY TO MEASURE THE CHAMFER ANYWAYS). FOR THIS REASON I DID NOT INCLUDE A TOLERANCE ON THE HEAD GEOMETRY.

PLEASE DEBURR THE CHAMFERED HEAD.

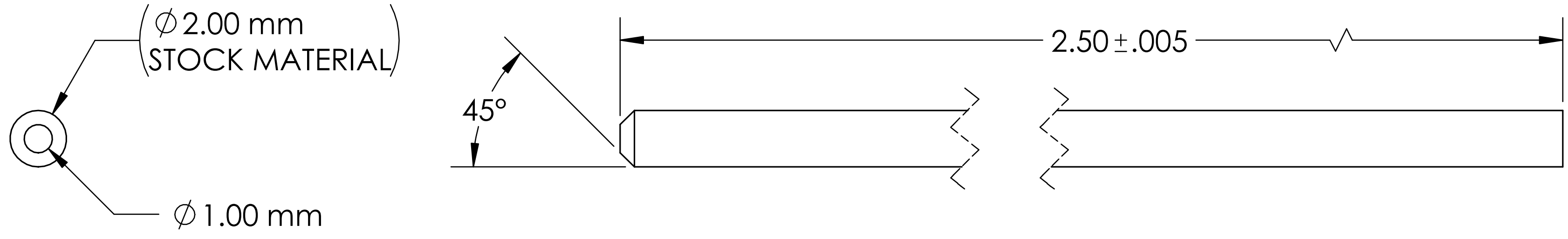

PROPRIETARY AND CONFIDENTIAL

THE INFORMATION CONTAINED IN THIS DRAWING IS THE SOLE PROPERTY OF Team 3 B.A.H. ANY REPRODUCTION IN PART OR AS A WHOLE WITHOUT THE WRITTEN PERMISSION OF Team 3 B.A.H, IS PROHIBITED.

DIMENSIONS ARE IN INCHES
THIRD ANGLE PROJECTION

MATERIAL: 17-4 Stainless Steel

STALK PUNCTURE NEEDLE

DEFAULT TOLERANCES:

LINEAR:	ANGULAR:
X. ±.25	X. ± 2
X.X ±.1	X.X ± 1
X.XX ±.01	X.XX ± 0 30'
X.XXX ±.002	

DESCRIPTION: Puncture Needle

AgMEQ LAB

CHECKED BY: XXXXXXXXXX DATE: XX/XX/XX

DRAWN BY: DATE: 2/9/2021

PART #: QTY:

FILE NAME: 00-02_SPUD.SLDPRT

SCALE: 4:1 SHEET: 1 OF 1
