# Supplementary figures and images for: Biomechanical phenotyping pipeline for stalk lodging resistance in maize
Source: MethodsX. 2024 Jan 9;12:102562. doi: 10.1016/j.mex.2024.102562 (PMC10825676; doi:10.1016/j.mex.2024.102562)

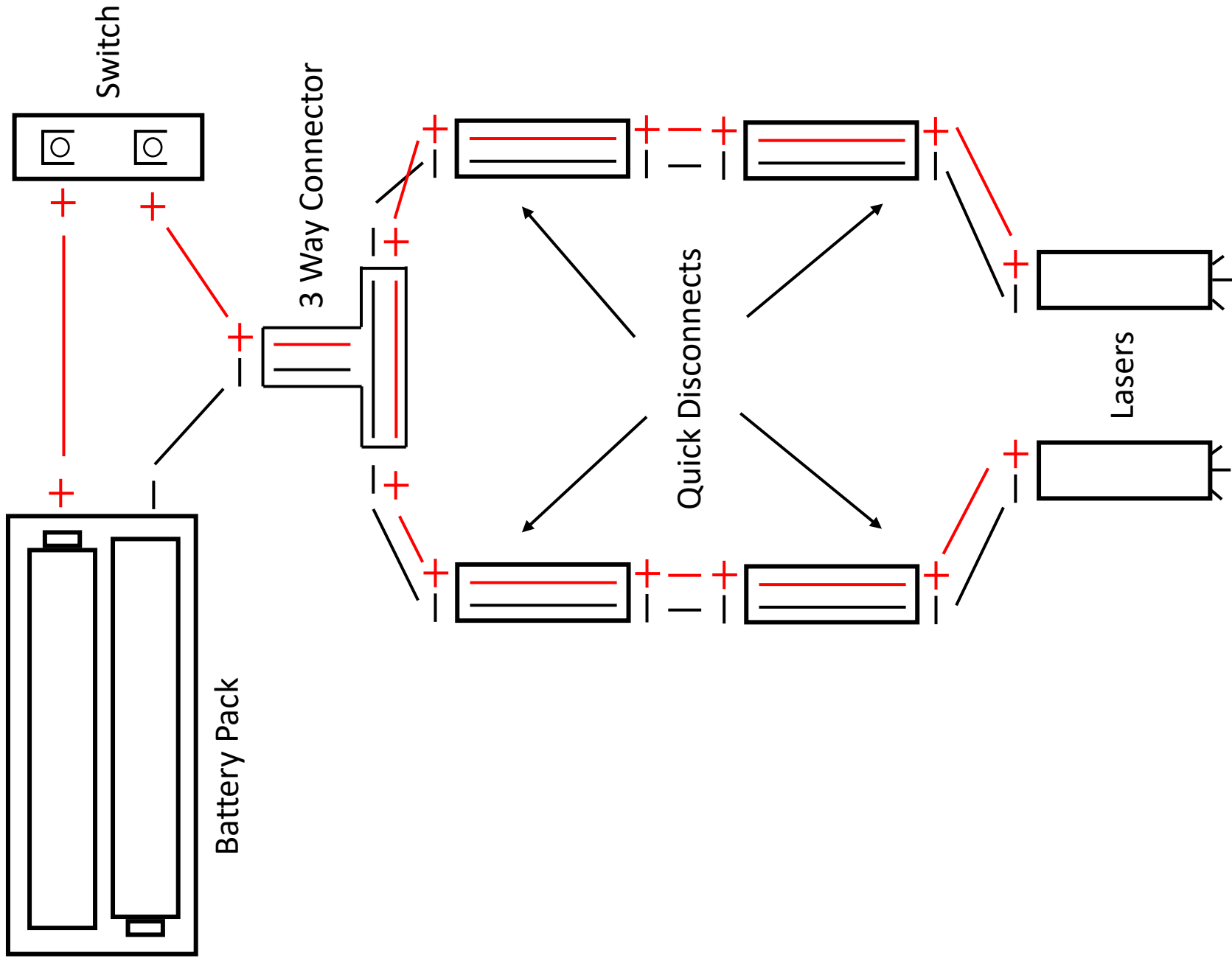

Supplement: Supplementary file 1 [file mmc1.zip › Supplimentary Material/RPR/Manufacturing Plans/Laser Sight/Wiring Diagram for Laser Sight.pdf]

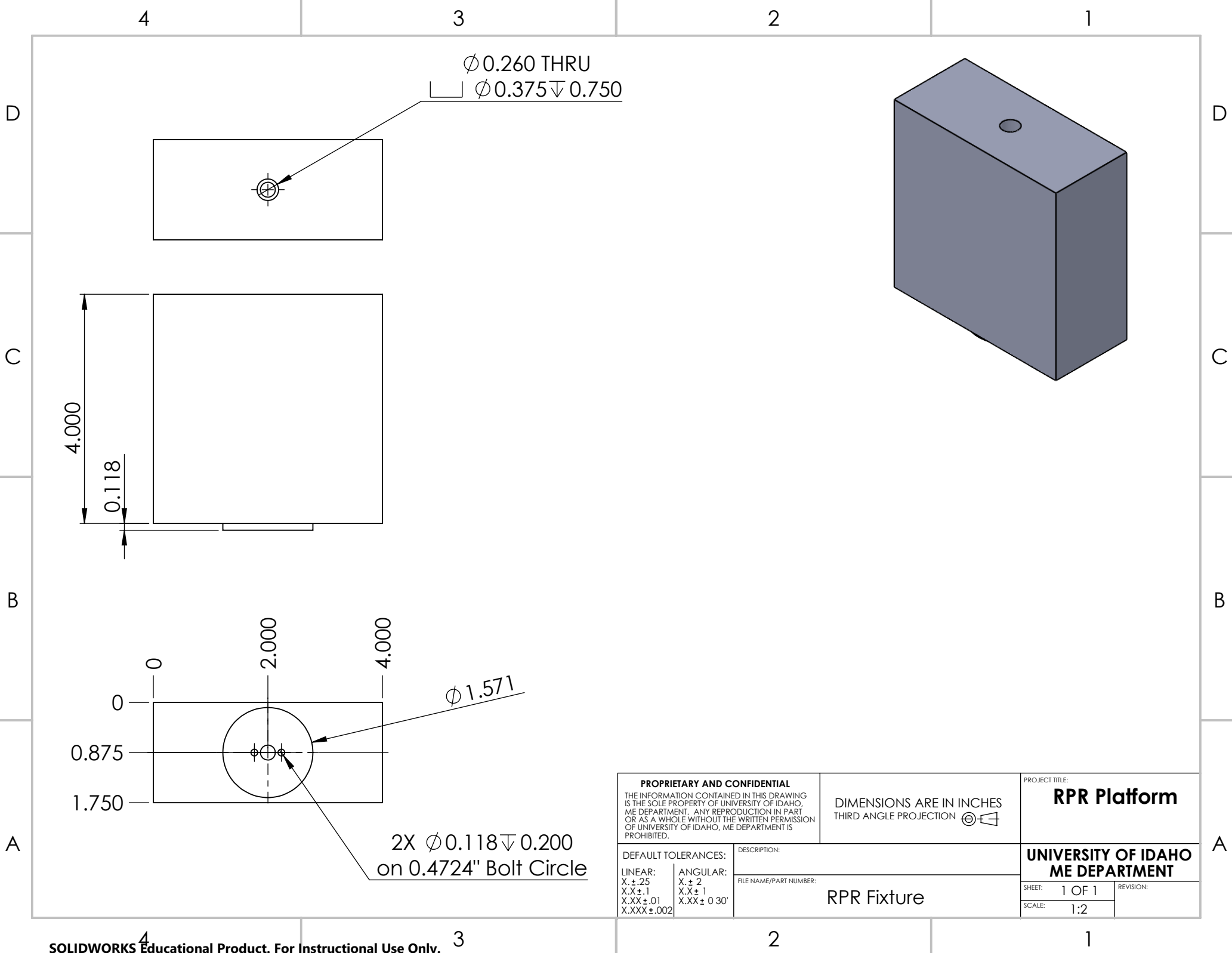

Supplement: Supplementary file 1 [file mmc1.zip › Supplimentary Material/RPR/Manufacturing Plans/RPR Platform/RPR Platform.pdf]
